# IGENT: efficient entropy based algorithm for genome-wide gene-gene interaction analysis

**DOI:** 10.1186/1755-8794-7-S1-S6

**Published:** 2014-05-08

**Authors:** Min-Seok Kwon, Mira Park, Taesung Park

**Affiliations:** 1 Interdisciplinary program in Bioinformatics, Seoul National University, Seoul, 151-747, Korea; 2 Department of Preventive Medicine, Eulji University, Daejeon, 301-768, Korea; 3 Department of Statistics, Seoul National University, Seoul, 151-747, Korea

## Abstract

**Background:**

With the development of high-throughput genotyping and sequencing technology, there are growing evidences of association with genetic variants and complex traits. In spite of thousands of genetic variants discovered, such genetic markers have been shown to explain only a very small proportion of the underlying genetic variance of complex traits. Gene-gene interaction (GGI) analysis is expected to unveil a large portion of unexplained heritability of complex traits.

**Methods:**

In this work, we propose IGENT, Information theory-based GEnome-wide gene-gene iNTeraction method. IGENT is an efficient algorithm for identifying genome-wide gene-gene interactions (GGI) and gene-environment interaction (GEI). For detecting significant GGIs in genome-wide scale, it is important to reduce computational burden significantly. Our method uses information gain (IG) and evaluates its significance without resampling.

**Results:**

Through our simulation studies, the power of the IGENT is shown to be better than or equivalent to that of that of BOOST. The proposed method successfully detected GGI for bipolar disorder in the Wellcome Trust Case Control Consortium (WTCCC) and age-related macular degeneration (AMD).

**Conclusions:**

The proposed method is implemented by C++ and available on Windows, Linux and MacOSX.

## Background

Recently, genome-wide association studies (GWAS) have been successful in understanding biological mechanisms and elucidating pathways that underlie complex genetic diseases [1]. However, GWAS were shown to explain only a small portion of the heritability of most complex diseases [2]. In order to find 'missing heritability' of complex diseases and understand genetic causes of diseases, gene-gene interaction (GGI) is expected to play an important role, because complex diseases are known to be controlled by multiple contributing genetic loci.

There are several statistical methods for detection of gene-gene interaction (GGI) [3]. One of conventional methods to characterize the interaction is regression analysis that includes main effects and relevant interaction terms. However, higher-order interaction may often cause the cell counts to be sparse, so that the parameter estimator may not be obtained. In order to avoid the sparsity problem in higher-order interaction, data mining methods such as support vector machine (SVM) and random forest (RF) were applied to find GGI. However, these methods could handle only a small number of variants due to their heavy computation [4,5].

The multifactor dimensionality reduction (MDR) method proposed by Ritchie *et al*. [6] is a non-parametric method that reduces the number of dimensions by converting a high-dimensional multi-locus model to a one-dimensional model to avoid the sparsity problem. MDR evaluates classifiers, which are SNP combinations associated with the disease of interest, to predict and classify disease status through cross-validation and permutation testing. The *k*-fold cross-validation splits the data into *k *subsets. The classifier is modelled on (*k*-1) subsets of the data and estimated by calculation of test accuracy on the remaining subset. This process is repeated for each subset. In addition to cross-validation, the permutation test can assess the statistical significance of MDR classifiers. However, it is unfeasible to use permutation tests for genome-wide scale interaction analysis because the permutation test is computationally intensive. To overcome this heavy computational burden, Pattin *et al*. proposed an efficient hypothesis test using extreme value distribution (EVD) [7]. Their simulation results showed that the proposed testing method requires at least 20 permutation data to keep up with similar power of 1000-fold permutation test.

Although MDR has a simple structure and fast computation, it is hard to find high-order interactions in large-scaled dataset because of its exhaustive searching scheme. For example, detection of 2nd order interactions for 300,000 SNPs requires computing 4.5 × 1010 combinations by MDR. When we use 10-fold cross-validation or 1000-fold permutation test, it takes 10 times or 1000 times longer.

Wan *et al*. proposed BOOST, which is a fast method for detecting gene-gene interaction using Boolean operation-based screening and testing [8]. BOOST is computationally efficient and detects statistical significant interactions based on approximated likelihood ratio statistic. Their simulation study showed that BOOST has higher statistical power than PLINK.

Recently, several approaches based on information theory for modelling GGI have been proposed [9-11]. Shannon started the information theory in 1948 by introducing the entropy that is measure for complexity in mathematical theory of communications [12].

Dawy *et al*. [9] proposed a relevance-chain method to identify the strongly associated lower-order interactions and build high-order interaction with the use of conditional mutual information. This method can provide fast detection of high-order interaction but it shows poor performance for GGI with no strong marginal effects. Chanda *et. al*. [10] proposed the k-way interaction information (KWII) metric and the total correlation information (TCI) for GGI identification. These entropy-based measures represent the amount of information of redundancy and dependency between SNPs and an environmental variable. This method performs a permutation test for statistical significance of detected interaction models. Ruiz-Marín *et al*. [11] proposed an entropy-based test for identification of single-locus association analysis. Although it showed a more powerful performance than the conventional Fisher tests, this method needs to be extended to handle GGI analysis. Yee *et al*. [13] proposed a modified entropy based method to evaluate the interactions between single SNP combinations. Their method was shown to be superior to the MDR method in most simulation cases. However, applying this entropy based method directly to the genome-wide scale data would be infeasible because of computationally intensive permutations.

In this paper, we develop a fast and efficient method, named IGENT, Information theory-based GEnome-wide gene-gene iNTeraction method, using entropy to identify the gene-gene interaction in genome-wide scale. IGENT supports two types of strategies to identify gene-gene interactions related with diseases in genome-wide scale. One is an exhaustive search approach for lower-order interactions such as 2nd order interaction, and the other is a stepwise selection approach for higher-order interaction. With tens of thousands of SNPs from thousands of samples, it is difficult to calculate higher-order interaction exhaustively because the computational burden is too heavy. IGENT provides a stepwise approach for higher-order interactions. The evaluation is based on the approximated gamma distribution of information gain without using permutation procedure, which allows us to overcome the computation burden for the GGI analysis in genome-wide scale [14].

## Methods

### Information theory

For detecting GGI associated with phenotypes, our measure is based on basic concept of information theory. The entropy, which measures the quantity of an uncertainty, is defined as

$$H\left(Y\right)=-\underset{j}{\sum }p\left(Y=y_{j}\right){\text{log}}_{2}p\left(Y=y_{j}\right),$$

where the entropy *H(X) *of a discrete random variable *Y *is a function of the probability distribution *p(Y=y_j_) *which measures the average amount of information contained in *Y*, or equivalently, the amount of uncertainty removed upon revealing the outcome of *Y*. Conditional entropy of *Y *given another discrete random variable *X *is

$$H\left(X\text{|}Y\right)=-\underset{i}{\sum }p\left(X=x_{i}\right)H\left(Y\text{|}X=x_{i}\right)$$

The information gain (IG) is defined as follows,

IG(Y|X)=H(Y)-H(Y|X)

IG which is also called mutual information (MI) can be explained as the reduction in entropy (or uncertainty) of one random variable given another. It is known that the IG follows gamma distribution with parameter *a *= (|*X*| − 1) (|*Y*| − 1) and *b *= 1/(*N *ln 2) approximately for the independent *X *and *Y *random variables [14].

(1) $$\hat{IG}\left(Y|X\right)\sim \text{Γ}\left(\frac{1}{2}\left(|Y|-1\right)\left(|X|-1\right),\frac{1}{N\text{ ln }2}\right)$$

where *N *is the sample size and |*X*| and |*Y*| denote the number of levels of the random variables *X *and *Y*.

## Entropy-based gene-gene interaction analysis

We use the information gain to detect GGI associated with phenotype. Given a case-control study with *n *individuals, let *Y *be the disease status and *X *be the SNP combinations, then

$$\begin{array}{c} H\left(Y\right)=H\left(disease\;status\right)\\ H\left(Y\text{|}X\right)=H\left(disease\;status\text{|}SNP\;combination\right) \end{array}$$

IG is given as

IG=H(Y)-H(Y|X)

The value of IG represents the true association strength. Since, under the null hypothesis of no association, IG follows a gamma distribution approximately by (1), we can assess the statistical significance of the association of SNP combinations and disease.

## Exhaustive searching approach and stepwise selection approach

**Figure 1:**
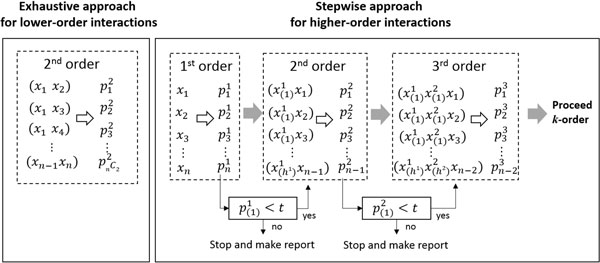
**Exhaustive approach and stepwise approach in IGENT**. t is threshold, $p_{j}^{k}$ is p-value for *j*^th ^combination in *k-*order interaction. $p_{\left(i\right)}^{k}$ is *i*^th ^ordered p-value among p-values of all combinations in *k-*order interaction. *h*^*k *^is the number of combinations over the threshold in *k*-order interaction.

We propose IGENT, an entropy-based gene-gene interaction method for genome-wide interaction analysis. IGENT supports exhaustive search (IGENT_exhaustive) for lower-order interaction and stepwise search (IGENT_stepwise) for higher-order interaction. In Figure 1, our exhaustive search approach and stepwise selection approach are described graphically.

IGENT_exhaust performs an exhaustive search for all possible combinations of variants for the given low order. IGENT_stepwise selects higher-order interactions in a stepwise manner. The detailed steps are summarized as the follows.

1. Initial step: for all SNPs, calculate 1^st ^order *IG^k ^*when k is order (in 1^st ^order, k = 1.).

2. Select SNP or SNP combinations with *p^k ^*<*t*, when *p^k ^*is p-value of hypothesis testing using the gamma distribution and *t *is significant threshold.

3. Calculate *IG*^*k*+1 ^for *k*+1 order interactions for the combinations with selected SNP or combinations adding additional other single SNP.

4. If there are significant interactions in *k*+1 order, *k *= *k *+ 1 and repeat step 2~4. Otherwise, stop forward addition and repeat 2~4 step with the next ranked combinations.

This IGENT_stepwise selection approach reduces search space dramatically. With large genome-wide scale data, this approach makes it feasible to discover higher-order interactions. Although this stepwise algorithm is not guaranteed to find the global optimum interaction model, it provides at least a local optimum interaction model with some marginal effects. Therefore, this stepwise approach may have a limitation in detecting the gene-gene interactions without any marginal effects.

## Implementation

Our method is implemented by C++ language. It is runnable on Windows, LINUX and MacOSX. This program supports both exhaustive search and stepwise search.

### Simulation studies

The main purpose of our method is to identify epistatic interactions from genome-wide data. In order to detect gene-gene interaction for genome-wide data, computational efficiency is a key issue. In simulation 1, we compared the computational efficiency of IGENT and other methods such as BOOST, MDR, RF and SVM. Among these methods, only IGENT and BOOST was shown to be feasible to analyze gene-gene interaction in genome-wide scale, as shown in simulation 1 of Results section. Thus, we mainly compared IGENT and BOOST in genome-wide scale with regard to the power of identifying causal gene-gene interaction through simulations 2, 3, and 4. In simulation 5, we compared IGENT_exhaust and IGENT_stepwise.

For these simulation studies, we use following three epistatic models:

1) Epistatic model set 1 : Eight interaction models

Models 1-1, 1-2, and 1-3 have different strength of genetic effects while fixing the interaction structure, the minor allele frequencies (MAF) and prevalence which have been used by Namkung *et al*. [15]. Models 1-4, 1-5, and 1-6 have different interaction structures and penetrance functions which were used by Ritchie *et al*. [16]. Models 1-7 and 1-8 were used by Bush *et al*. [17]. Eight interaction models are summarized in additional file 1.

2) Epistatic model set 2 : four interaction models with main effects

Model 2-1 is a multiplicative model. Model 2-2 is an epistasis model that has been used to describe handedness and the colour of swine. Model 2-3 is a classical epistasis model. Model 2-4 is the XOR model. The details of these four models have been described by Wan *et al*. [8].

3) Epistatic model set 3 : Seventy interaction models without main effects

Seventy Disease models without main effects have been proposed by Velez *et al*. [18]. These 70 epistatic models are distributed across six heritability values (0.01, 0.025, 0.05, 0.1, 0.2, and 0.4) and two different MAFs (0.2 and 0.4).

Using these epistatic model sets, we conduct the following five simulation studies.

#### Simulation 1: comparing computational efficiency for genome-wide gene-gene interaction analysis

To compare computational efficiency with IGENT, BOOST, MDR, SVM and RF, we construct simulation data using the epistatic model set 1. Each epistatic models contains 2000 individuals balanced between cases and controls. Various numbers of SNPs (50, 100, 500, 1K, 2K, 5K, 10K, 100K, 350K, and 500K) are considered. All analysis are carried out on single core of a 3.16 GHz CPU with 4G memory on LINUX.

#### Simulation 2: estimating type I error in null simulation

To take an assessment in terms of type I error, we construct 1000 replicates of null simulation data with 1000 SNPs and 1000 individuals based on the epistatic model set

1. In this null simulation data, all SNPs have no association with disease status. Using null simulation, we compare false positive rates of IGENT and BOOST.

#### Simulation 3: comparing the power of gene-gene interaction with main effects

To compare the power of IGENT and BOOST in gene-gene interaction with main effects, we use the epistatic model set 2. The MAFs of disease-associated SNPs is set to be 0.1, 0.2, and 0.4. Each data set has 1000 SNPs from 800 and 1600 individuals. We generate 100 replicate data sets under each setting. Using this simulation, we compare the power of IGENT and BOOST for gene-gene interaction with main effects.

#### Simulation 4: comparing the power of gene-gene interaction without main effects

For evaluation of finding causal gene-gene interaction with no marginal effects, we use the epistatic model set 3. Using these 70 epistasis models in the set, we generate 100 replicate sets with 1000 SNPs (one pair is causal interaction, others are non-causal SNPs), and four sample sizes (200, 400, 800, and 1600 individuals).

#### Simulation 5: comparing the efficiency of stepwise search approach

For comparison of the efficiency of IGENT_stepwise, we use the epistatic model set 1. We generate 100 replicate set with 50 SNPs from 400 individuals. Through this simulation, we compare the power and computational efficiency of IGENT_stepwise and IGENT_exhaust.

## Genome-wide data

### Bipolar disorder (BD) data analysis

Using bipolar data from the Wellcome Trust Case Control Consortium (WTCCC) [19], we demonstrated genome-wide gene-gene interaction analysis for 2^nd^-order and higher-order interaction. SNPs with call rates <95% were excluded from the analysis. SNPs showing Hardy-Weinberg equilibrium (HWE) p-value<5.7 × 10^-7 ^were filtered out. Of the remaining SNPs, only SNPs showing MAF of at least 5% were carried forward for further analysis. All quality control steps were conducted using PLINK version 1.07 [20] and R scripts. We performed imputation using fastPHASE version 1.2 [21] to increase the density of interrogated SNPs. After quality control and imputation process, WTCCC-BD dataset contained 354,022 SNPs and 4,806 samples.

IGENT was applied for exhaustive two-way interaction analysis of 6.27 × 10^10 ^pairs of SNPs for WTCCC-BD data and stepwise selection approach for higher-order interactions.

#### Age-related macular degeneration (AMD) data analysis

For real data application, we used the AMD data set which contains 116,209 SNPs genotyped with 96 cases and 50 controls from the Age-Related Eye Disease Study (AREDS) [22]. We conducted the same quality control process as in the BD data analysis except forMAF < 0.01. All quality control steps were conducted using PLINK version 1.07 [20] and R scripts. After quality control process, we used remained 102,504 SNPs from 146 individuals. Pair-wise interaction analysis of all 5,253,483,756 pairs was conducted with IGENT_exhaust and BOOST. Also, IGENT_stepwise was performed for higher-order interactions.

## Results

### Simulation results

In this section, we perform simulation studies to evaluate the properties of IGENT and to compare it with other previous proposed methods. In order to detect gene-gene interaction with genome-wide data, computational efficiency is a key issue. In simulation 1, we compared the computational efficiency of IGENT and other methods such as BOOST, MDR, RF, and SVM. Among these methods, only IGENT and BOOST were shown to be feasible to analyze gene-gene interaction in genome-wide scale in simulation 1. We mainly compared IGENT and BOOST in regard to the power of identifying causal gene-gene interaction in simulations 2, 3, and 4. In simulation 5, we compared IGENT_stepwise and IGENT_exhaust.

#### Simulation 1: comparing computational efficiency for genome-wide gene-gene interaction analysis

In order to compare the computational efficiency of IGENT and other methods including BOOST, MDR, RF, and SVM, we conducted 2^nd ^order interaction analysis with various the number of SNPs (50 to 500K). We used LIBSVM library [23] and "randomforest" R package [24] for SVM and RF methods, respectively. All methods used an exhaustive search strategy for fair comparison.

Table 1 presents computation times to finish 2^nd ^order interaction analysis by each method. In simulation data with 350K SNPs, IGENT_exhaust and BOOST can finish the interaction analysis within about 2.17 days and 1.8 days, respectively. However, due to their heavy computation times, MDR, RF, and SVM are not feasible to conduct the gene-gene interaction analysis with genome-wide dataset. For focusing on genome-wide interaction analysis, we thus compare the power of IGENT and BOOST in simulations 2, 3, and 4.

**Table 1:** Computation time of IGENT, BOOST, MDR, RF, and SVM.

SNP size	IGENT_exhaust	BOOST	MDR	RF	SVM
50	<1s	<1s	1s	11s	13s
100	<1s	<1s	4s	46s	53s
500	<1s	<1s	1m 8s	20m	23m
1K	3s	1s	4m 25s	1h 15m	1h 29m
2K	8s	6s	19m 52s	5h	5h 50m
5K	38s	30s	2h 4m	1d 6h	1d 12h
10K	2m 34s	2m 7s	*8h 16m	*5d 5h	*6d 3h
100K	4h 23m	3h 32m	*35d	*520d	*614d
350K	2d 4h	1d 19h	*422d	*6366d	*7524d
500K	4d 10h	3d 15h	*861d	*12992d	*15353d

Computation time is measured in simulation 1 dataset which have 2000 individuals. All methods used an exhaustive search strategy for 2nd order interaction analysis.

* This computing time is estimated from the computing time in simulation data with 5000 SNPs.

All analysis are carried out on single core of a 3.16 GHz CPU with 4G memory on LINUX.

#### Simulation 2: estimating type I error in null simulation

The type 1 error rates of IGENT_exhaust and BOOST are shown in Table 2. Although the type I error rates of IGENT_exhaust and BOOST seem to be slightly higher than the nominal value, it can be shown that the type I errors of IGENT and BOOST agree with the nominal value lying within the confidence interval.

**Table 2:** Comparison of the type I error in null simulation

**Thresholds after Bonferroni correction**	False Positive Rate	
	
	IGENT	BOOST
0.01	0.012	0.011
0.05	0.057	0.054
0.10	0.112	0.108
0.15	0.166	0.153
0.20	0.219	0.215
0.25	0.270	0.264
0.30	0.321	0.318

#### Simulation 3: comparing the power of gene-gene interaction with main effects

**Figure 2:**
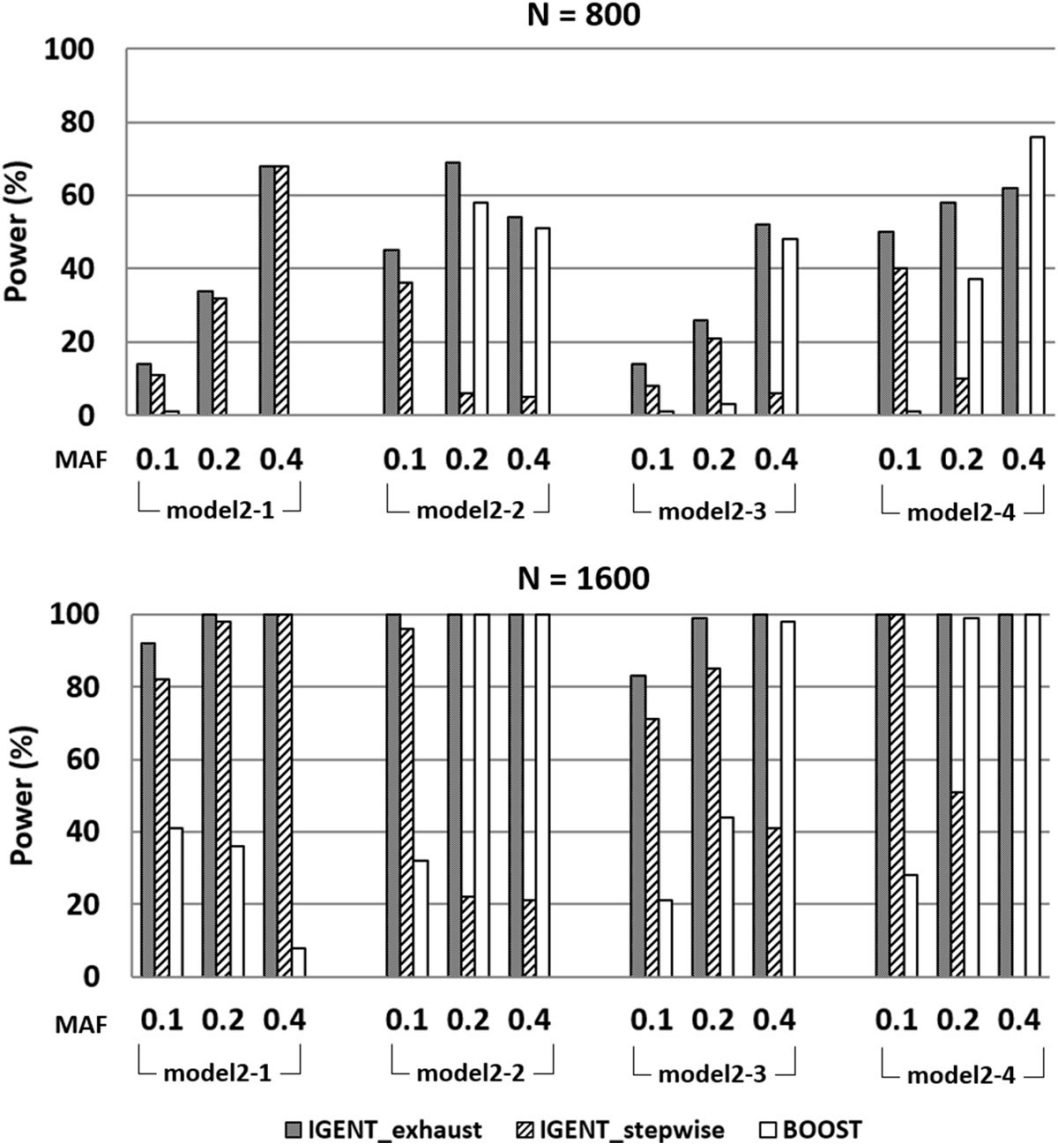
**The power comparison between IGENT and BOOST on four disease models with main effects**. Results are shown in separate panels for each sample size (800 and 1600). MAF are presented on the x-axis. Model 2-1 is a multiplicative model. Model 2-2 is an epistasis model that has been used to describe handedness and the colour of swine. Model 2-3 is a classical epistasis model. Model 2-4 is the XOR model.

In simulation 3, we compared the IGENT_exhaust, IGENT_stepwise, and BOOST for detecting causal gene-gene interactions with main effects. In simulation data, IGENT used both exhaustive mode and stepwise mode, and BOOST used an exhaustive mode for searching the 2^nd ^order interactions. The power is calculated as the proportion of 100 data sets in which the interactions of the disease-associated SNPs are detected. In all simulation data, we counted the interaction with its p-value (after multiple comparison procedure by Bonferroni correction) < 0.05. In stepwise mode, only variants with marginal p-value < 0.05 were proceeded to the next step for calculating 2^nd ^order interactions. In simulation 3, the detection probability of IGENT_exhaust showed the best performance in most models except for Models 2-4 (Figure 2). The performance of BOOST became worse in the simulation models with low minor allele frequency (MAF 0.1 and 0.2). In simulation 3, the average power of IGENT_stepwise was about 60% relative to IGENT_exhaust, but its computing time was less than 1%(only 0.43%) of IGENT_exhaust.

#### Simulation 4: comparing the power of gene-gene interaction without main effects

**Figure 3:**
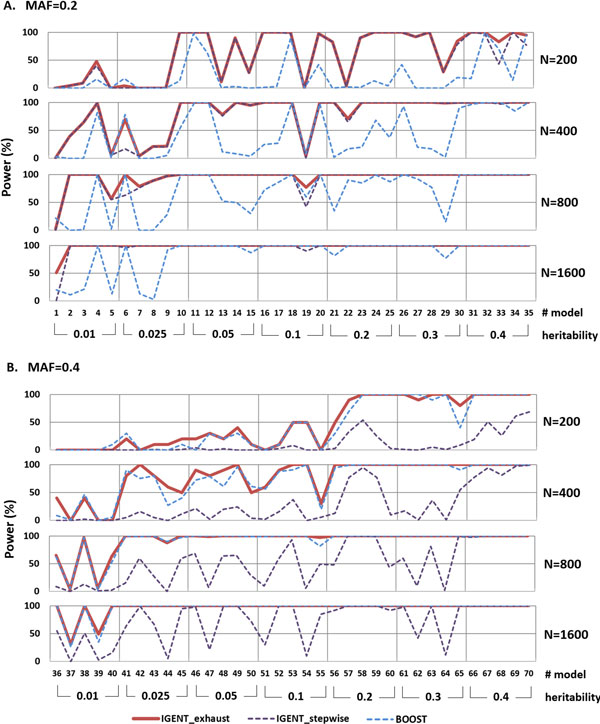
**Performance comparison with IGENT, BOOST in 70 simulation models**.

In simulation 4 which has causal gene-gene interaction without main effects, IGENT_exhaust performed better than or equivalent to BOOST in most simulation models. In simulation model with lower MAF and small sample size, BOOST showed poor performance. However, they provided equivalent results for models with a MAF of 0.4 or large sample sizes (Figure 3).

#### Simulation 5: comparing the efficiency of stepwise analysis and exhaust analysis of IGENT

We evaluated the performance of IGENT_stepwise in simulation 5 based on epistatic model set 1. All models were designed with the 2^nd ^order interaction effects and no marginal effects. Although these simulation models do not include the higher-order interaction effects over the 2^nd ^order, it is possible for spurious higher-order interaction to show the large effects on phenotype. To allow for finding spurious higher-order interactions, we exhaustively identified interactions from 1^st ^to 4^th ^orders. By comparing the identified interactions from IGENT_exhaust to those from IGENT_stepwise, we were able to evaluate the performance of IGENT_exhaust.

Table 3 shows IGENT_stepwise has the 66~93% of power of the IGENT_exhaust by using only 12~36% computation of the IGENT_exhaust. For the genome-wide interaction analysis, IGENT_stepwise can perform high-order interaction analysis very efficiently.

**Table 3:** Efficiency of stepwise analysis

Model	Power^a ^in Stepwise approach	Power in exhaustive approach	ratio of power^b^	Computation in stepwise approach^c^	ratio of computation^d^
**1**	0.69	1.00	**0.69**	148.4	**0.12**
**2**	0.71	0.92	**0.77**	149.7	**0.12**
**3**	0.67	0.80	**0.84**	154.7	**0.13**
**4**	0.87	0.94	**0.93**	147.6	**0.12**
**5**	0.62	0.88	**0.70**	147.0	**0.12**
**6**	0.63	0.96	**0.66**	145.3	**0.12**
**7**	0.19	0.25	**0.76**	167.3	**0.14**
**8**	0.15	0.17	**0.88**	445.6	**0.36**

^a ^Detection probability,

^b ^the ratio of power between stepwise approach and exhaustive approach

^c ^Average number of combinations to be computed in stepwise approach

^d^Computation ratio is the ratio of computation amount of stepwise approach and computation amount of exhaustive approach. The computation of exhaustive approach is calculated using _2_C_50 _= 1225.

## Analysis of real data: WTCCC bipolar disorder (BD) data

We conducted genome-wide two-way interaction analysis and higher-order interactions with WTCCC-BD dataset [19]. The IGENT_exhaust completed all two-way interaction pairs (6.25 × 10^9^) in about 74 hours on a 3.16 GHz CPU with 4G memory on LINUX. IGENT_stepwise took about 1.5 hour in higher order interactions on the same system. Through exhaustive two-way interactions, IGENT_exhaust reported 39 significant interactions. Among these 39 interactions, 26 pairs were also reported by IGENT_stepwise. Among these hub genes, LOC390730, DPP10, and CDC25B have been reported with strong marginal effects in a previous study [19] (Table 4). B2GALT5, PI15, TLE4, AKAP10, and CHST2 did not show significant associations in single locus analysis but showed strong interactions. These genes have been reported as causal genes associated with bipolar disorder in other studies [25-30].

**Table 4:** Hub genes (degree of nodes ≥ 10) in two-way interactions of WTCCC-BD

Hub gene	degree	location	SNP(s)	Reference^a ^
B3GALT5	115	21q22.2b	rs980184	[25]
LOC442261	98	6q23.2d	rs4896044	
PI15	32	8q21.11b	rs2954873	[26]
LOC390730	26	16q12.2a	rs7188309 rs11640993 rs8056052 rs2192859 rs1344484 rs10521275 rs11647459 rs2387823	[19]
PHF20	24	20q11.23a	rs6060710	
TLE4	13	9q21.31b	rs914715 rs11138278	[27]
DPP10	12	2q14.1b	rs11123306 rs708647 rs1375144 rs6741692	[28,19]
AKAP10	10	17p11.2d	rs203466 rs203457 rs119672 rs2108978	[29]
CHST2	10	3q23d	rs4683457	[30]

a. Reference is literature related with bipolar disorder.

**Figure 4:**
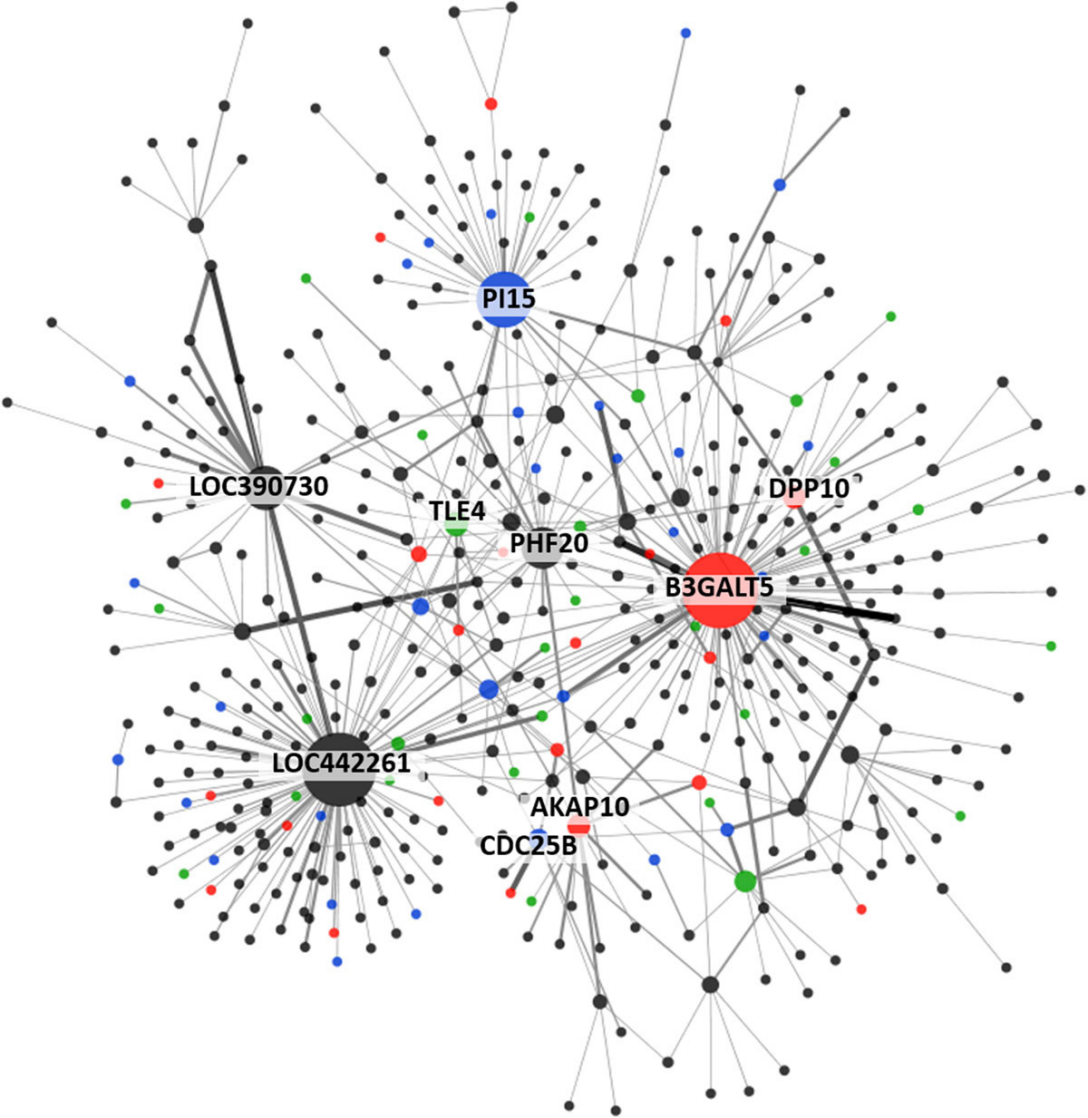
**Gene-gene interaction network for WTCCC-BD dataset**. Red nodes represent genes reported in previous GWAS literature with bipolar disorder dataset. Blue nodes are the genes related with bipolar disorder in previous literature. Green nodes are the genes related with other psychiatric disorders (schizophrenia and depression disorder). Width of edge is the significance level of interaction.

In Figure 4, using two-way interaction analysis by IGENT, we constructed the interaction network of WTCCC-BD. In two-way interaction network, a node represents a gene with SNP(s), edge is interaction reported by IGENT analysis. Node size shows the degree of the node and edge width shows the number of SNP-SNP interactions. All significant interactions were annotated by HuGE navigator database [31] and GWAS catalog [32]. This network graph represents two-way interactions of genome-wide association with bipolar disorder and facilitates biological interpretations.

## Analysis of real data: AMD data

We conducted 2^nd ^order interaction analysis and high-order interaction analysis using IGENT and BOOST for AMD data. Table 5 shows the top 5 interactions or SNP identified by IGENT. In the case of AMD data, there are SNPs (rs380390 (CFH) and rs1329428 (CFH)) with strong marginal effect. These SNPs were also reported previously that they have strong association with AMD disorder [22]. IGENT also detected two interactions (CFH (rs380390) - SGCD (rs931798) and CFH (rs1329428) - MED27 (rs9328536)). These two interactions also have a SNP with a strong marginal effect.

**Table 5:** Interaction analysis result using AMD data set

rank	SNP	*P*
1	CFH(rs380390) SGCD(rs931798)	8.454 × 10^-12^
2	CFH(rs1329428) MED27(rs9328536)	1.943 × 10^-10^
3	CFH(rs380390)	2.087 × 10^-7^
4	INPP4B(rs3775640)	3.128 × 10^-7^
5	CFH(rs1329428)	1.166 × 10^-6^

## Discussion

In this paper, we proposed a fast analysis for searching for high-order interactions associated with complex diseases. IGENT uses information gain which represents association strength with GGI and phenotype without a specific genetic model. The IG measure can be used to compare the association strength across different order of interactions. IGENT adopts an exhaustive search scheme that investigates all possible interactions in lower-order interactions and a stepwise search scheme for higher-order interactions. The permutation and exhaustive search schemes of the previous GGI methods are computationally too intensive to be employed in large genome-wide scale data set for high-order interactions.

Note that IGENT is as fast as BOOST and shows better performance than BOOST. BOOST has been known to have a limitation that the degree of freedom of the statistical test should be reduced when the contingency table is too sparse due to low MAF [8]. IGENT, however, presents stable performance in various epistasis models even with low MAF.

To evaluate significance of IGENT's result, we used hypothesis testing framework by approximating the gamma distribution. It is known that IG follows the gamma distribution under the null hypothesis. Using approximation to the gamma distribution instead of permutation, we can easily calculate statistical significant interactions and save the computation time remarkably. A stepwise approach is more efficient than exhaustive approach in terms of computation. However, this stepwise approach has a trade-off between computational efficiency and detection of optimal gene-gene interactions. Our stepwise approach, IGENT_stepwise, reduced a search space extremely for detecting GGI with marginal effects. Although GGI without marginal effects can be generated mathematically [33-35], it is still unclear in practice how the GGI model without marginal effect is biologically associated with a complex disease [3].

In an exhaustive search scheme, our simulation result showed that IGENT_exhaust consistently had better performance than BOOST, as shown in Figures 2 and 3. Although both BOOST and IGENT showed efficient and fast computational performances, IGENT showed power higher than or equivalent to that of BOOST.

## Conclusions

In conclusion, we proposed a fast and efficient enhanced entropy-based GGI analysis method. Due to its fast and efficient computation scheme, it can easily identify the gene-gene interaction in genome-wide scale. Through real GWAS data analysis, IGENT successfully identified low order and high order interactions. IGENT has been implemented with C++, and is available in http://bibs.snu.ac.kr/software/igent.

## List of abbreviations used

IGENT, Interactions analysis method in Genome-wide scale based on ENTropy; WTCCC, the Wellcome Trust Case Control Consortium; BD, Bipolar disorder; SVM, support vector machine; RF, random forest; GGI, gene-gene interaction; IG, information gain;

## Competing interests

The authors declare that they have no competing interests.

## Authors' contributions

MK performed the programing and the analysis, and drafted the manuscript. MP participated in the design of the study. TP conceived of the study, and participated in its design and coordination and helped to draft the manuscript. All authors write, read and approved the final manuscript.

## Supplementary Material

Additional file 1 Eight epistatic interaction models used in simulation 3. Additional file descriptions text (including details of how to view the file, if it is in a non-standard format). Click here for file

## Declarations

Publication for this article has been funded by the Seoul National University.

This article has been published as part of *BMC Medical Genomics *Volume 7 Supplement 1, 2014: Selected articles from the 3rd Translational Bioinformatics Conference (TBC/ISCB-Asia 2013). The full contents of the supplement are available online at http://www.biomedcentral.com/bmcmedgenomics/supplements/7/S1.
