# Supplementary material for: IGENT: efficient entropy based algorithm for genome-wide gene-gene interaction analysis
Source: BMC Med Genomics. 2014 May 8;7(Suppl 1):S6. doi: 10.1186/1755-8794-7-S1-S6 (PMC4101351; doi:10.1186/1755-8794-7-S1-S6)
Supplement: Additional file 1 — Eight epistatic interaction models used in simulation 3. Additional file descriptions text (including details of how to view the file, if it is in a non-standard format). [file 1755-8794-7-S1-S6-S1.docx]

**Additional file 1**

|  | **Model 1** | | |  | **Model 2** | | |  | **Model 3** | | |  | **Model 4** | | |
| --- | --- | --- | --- | --- | --- | --- | --- | --- | --- | --- | --- | --- | --- | --- | --- |
| Prevalence | 0.05 | | |  | 0.05 | | |  | 0.05 | | |  | 0.046 | | |
| MAF | 0.1 | | |  | 0.1 | | |  | 0.1 | | |  | 0.1 | | |
|  | **AA** | **Aa** | **aa** |  | **AA** | **Aa** | **aa** |  | **AA** | **Aa** | **aa** |  | **AA** | **Aa** | **aa** |
| **BB** | 1.21 | 0.20 | 0.20 |  | 1.23 | 0.33 | 0.33 |  | 1.22 | 0.40 | 0.40 |  | 0.55 | 1.75 | 1.33 |
| **Bb** | 0.20 | 5 | 5 |  | 0.33 | 3 | 3 |  | 0.40 | 2.50 | 2.50 |  | 1.54 | 0.18 | 0.74 |
| **bb** | 0.20 | 5 | 5 |  | 0.33 | 3 | 3 |  | 0.40 | 2.50 | 2.50 |  | 1.75 | 0.18 | 0 |
|  |  |  |  |  |  |  |  |  |  |  |  |  |  |  |  |
|  |  |  |  |  |  |  |  |  |  |  |  |  |  |  |  |
|  | **Model 5** | | |  | **Model 6** | | |  | **Model 7** | | |  | **Model 8** | | |
| Prevalence | 0.026 | | |  | 0.017 | | |  | 0.052 | | |  | 0.048 | | |
| MAF | 0.1 | | |  | 0.1 | | |  | 0.2 | | |  | 0.4 | | |
|  | **AA** | **Aa** | **aa** |  | **AA** | **Aa** | **aa** |  | **AA** | **Aa** | **aa** |  | **AA** | **Aa** | **aa** |
| **BB** | 1.16 | 0.38 | 0.76 |  | 1.15 | 0.40 | 0.17 |  | 0.84 | 1.35 | 0.80 |  | 0.52 | 1.07 | 1.89 |
| **Bb** | 0.38 | 3.70 | 1.97 |  | 0.28 | 4.23 | 4.89 |  | 1.30 | 0.39 | 1.45 |  | 1.30 | 0.92 | 0.59 |
| **bb** | 0.76 | 1.97 | 2.92 |  | 1.15 | 0.06 | 5.56 |  | 1.45 | 0.13 | 1.04 |  | 1.21 | 1.08 | 0.33 |
